# Genome of *Pediococcus acidilactici* ITV26 isolated from neonatal feces: enzymatic potential for food industrial applications

**DOI:** 10.1128/mra.00575-26

**Published:** 2026-09-30

**Authors:** Karla Gabriela Álvarez-Villagómez, Carolina Peña-Montes, Itzel del Carmen Fonseca-Barrera, Micloth López-Del-Castillo-Lozano, Patricia Guillermina Mendoza-García

**Affiliations:** 1 Unidad de Investigación y Desarrollo en Alimentos (UNIDA), TecNM/Instituto Tecnológico de Veracruz, Veracruz, México; 2 Universidad Veracruzana, Instituto de Ciencias Básicas, Veracruz, México; Portland State University, Portland, Oregon, USA

**Keywords:** *Pediococcus acidilactici*, antimicrobial activity, probiotic potential, food safety, whole-genome sequencing, flavor enhancer

## Abstract

The genome of *Pediococcus acidilactici* ITV26, isolated from neonatal feces, comprises 1,961,346 bp and is 99.8% complete. Annotation identified 1,930 genes, including specific peptidoglycan hydrolase with antimicrobial activity, probiotic-associated proteins mediating intestinal adhesion and acid tolerance, and enzymes predicted to drive flavor development in fermented foods, confirming its multifunctional biotechnological potential.

## ANNOUNCEMENT

*Pediococcus acidilactici* is a food-grade lactic acid bacterium with potential for biotechnological applications due to its enzymatic production of bioactive peptides and volatile precursors (1). Furthermore, it harbors peptidoglycan hydrolases (PGHs) (2).

*P. acidilactici* ITV26 was isolated from infant fecal samples (0–6 months) donated in (August 1995) at Daycare N°1 of the Instituto Mexicano del Seguro Social (IMSS), Veracruz, Mexico. Samples were anaerobically enriched in de Man, Rogosa, and Sharpe (MRS) broth (37°C, 24 h) and plated on MRS agar, and isolates were confirmed by Gram staining, catalase testing, and colony morphology. Initial identification was based on biochemical analysis and 16S ribosomal RNA (rRNA) gene sequencing, showing 98.23% identity with *P. acidilactici* NGRI 0510Q (Accession no. NR_041640.1) (3). Following evaluation of its antimicrobial properties (2–4), the strain was subjected to whole-genome sequencing. Genomic DNA was extracted from 1.8 mL of culture grown in MRS broth at 37°C for 12 h without agitation. Cells were harvested at the logarithmic growth phase (OD_600_ = 0.85 ± 0.04) using the AllPrep Bacterial DNA/RNA/Protein Kit (Qiagen, Hilden, Germany). DNA purity was assessed by the *A*_260_*/A*_280_ ratio using a BioTek Epoch two spectrophotometer (BioTek Instruments, USA). The library was prepared using the SQK-RAD114 rapid sequencing kit and sequenced on an R10.4.1 (FLO-MIN114) flow cell using the MinION platform (Oxford Nanopore Technologies, Oxford, UK), targeting a minimum sequencing depth of 30×. Basecalling was performed with Dorado v0.7.3 using the super-accuracy model (dna_r10.4.1_e8.2_400bps_sup@v4.3.0), retaining reads with a minimum Q-score of 10 and a minimum length of 200 bp. *De novo* assembly with Flye v2.9.4, followed by polishing with Medaka v2.0.0, achieved a mean genome coverage of 675×. Genome completeness and contamination were assessed using CheckM (v1.2.2) via the Bacterial and Viral Bioinformatics Resource Center (BV-BRC) platform. The assembled genome of ITV26 comprises 1,961,346 bp and is resolved into two contigs, a 1.91 Mb chromosome and a 48.9 kb plasmid. Functional annotation was performed using the National Center for Biotechnology Information (NCBI) Prokaryotic Genome Annotation Pipeline (v6.11). The general genomic features are summarized in Table 1.

**TABLE 1:** Genomic features of *P. acidilactici* ITV26^*a*^

Features	Values
Read quality	
Total reads	270,311
Total bases	~1.35 Gb
Mean read length	4,992 bp
Median read length	3,203 bp
Read N50	8,409 bp
Assembly and quality	
Completeness	94.01%
Contamination	1.77%
Genome features	
Genome size (bp)	1,961,346
No. of contigs	2
Chromosome size	1.91 Mb
Plasmid size	48.9 kb
Contig N50	1,912,381 bp
G + C content	42.5%
Annotation	
Total genes	1,930
Protein-coding sequences	1,787
RNA genes	76 (57 tRNAs, 15 rRNAs, 4 ncRNAs)
Pseudogenes	67
CRISPR arrays	1
Accession numbers	
BioProject	PRJNA1453559
SRA accession	SRR38396699
GenBank accession	JBXOWU000000000.1

^*a*^ bp, base pairs; Gb, gigabases; kb, kilobases; Mb, megabases; tRNA, transfer RNA; rRNA, ribosomal RNA; ncRNA, non-coding RNA.

The genome of *P. acidilactici* ITV26 encodes four PGHs with putative lytic activity (5–7) that remain to be experimentally characterized: two GH25 family lysozymes (MHR6003731.1 and MHR6004203.1), an N-acetylmuramoyl-L-alanine amidase (MHR6004229.1), and a GH73 family protein (MHR6002763.1). Notably, our group has already characterized the glucosaminidase domain-containing protein (MHR6003589.1), which demonstrated potent antibacterial activity against both gram-negative and gram-positive pathogens in experimental assays (2). The genome also harbors determinants relevant to its probiotic potential, including a putative mucin-binding protein and predicted arginine deiminase (*arcA*), as well as genes associated with flavor development such as a malolactic enzyme (*mle*) and *alsS-budA* diacetyl/acetoin biosynthetic cluster (8, 9). Since these functions are based on genomic annotation, they are considered putative activities that warrant further experimental validation. Characterization of the mucin-binding protein and *arcA* gene identified here, including their functional and phenotypic validation, will be presented in a companion manuscript (K. G. Álvarez-Villagómez, E. T. Santos-Caiceros, C. Peña-Montes, M. López-Del-Castillo-Lozano, D. Balleza , P. G. Mendoza-García, unpublished data).

These findings suggest the possible enzymatic multifunctionality of *P. acidilactici* ITV26, supporting its confirmed antimicrobial activity and its possible potential probiotic and functional food applications, as well as for flavored fermented products.

## Data Availability

This Whole Genome Shotgun project has been deposited at DDBJ/ENA/GenBank under the accession JBXOWU000000000.1. The raw sequence reads were deposited in the NCBI Sequence Read Archive (SRA) under BioProject accession number PRJNA1453559 (SRA accession SRR38396699).
